# Affordable *Caenorhabditis elegans* tracking system for classroom use

**DOI:** 10.17912/micropub.biology.000377

**Published:** 2021-03-17

**Authors:** Nicholas Leonard, Andrés G. Vidal-Gadea

**Affiliations:** 1 Normal University High School, Normal, Illinois; 2 School of Biological Sciences, Illinois State University, Normal, Illinois

## Abstract

For decades the nematode *C. elegans* has served as an outstanding research organism owing to its unsurpassed experimental amenability. This advantage has also made this tiny worm an attractive vehicle for science instruction across higher learning institutions. However, the prohibitive cost associated with the automated behavioral assessment of these animals remains an obstacle preventing their full adoption in undergraduate and high school settings. To improve this situation, we developed an inexpensive worm tracking system for use by high school interns and undergraduate students. Over the past two years this tracker has been successfully used by undergraduate students in our introductory Cell and Molecular lab (BSC220) at Illinois State University. Here we describe and demonstrate the use of our inexpensive worm tracking system.

**Figure 1. Construction and use of low cost Caenorhabditis elegans tracking system:**
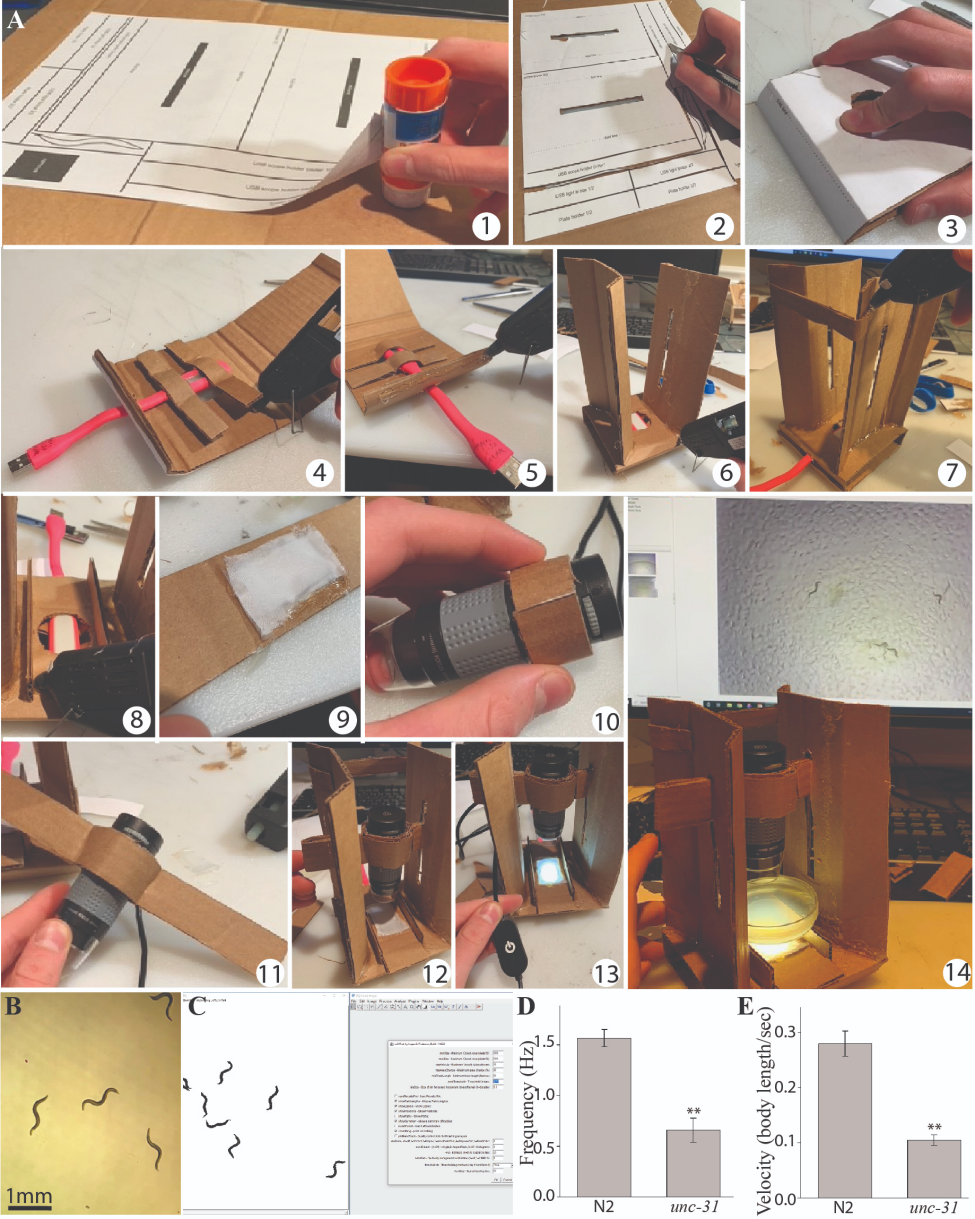
**A)** Assembly of the worm tracker takes under one hour (1-14). See step by step instructions below. **B)** Sample frame from movie obtained with the tracker. **C)** Movie processing with the wrMTrck plugin for ImageJ. We filmed and compared swimming in day 1 adult wild-type (N2) and *unc-31(e928)* mutants to illustrate the capability of the tracking system. Four swimming assays with eight animals each were performed for each strain. **D)** The synaptic mutant *unc-31(e928)* has significant impairments in swimming frequency and normalized (to body length) velocity **(E)** compared to N2 worms. ** p<0.001, two-tailed t-test.

## Description

From the beginning of *Caenorhabditis elegans*’ inception as a genetic model organism by Sydney Brenner (Brenner 1974), the ability to measure and quantify behavior in these nematodes led to numerous and powerful insights (Apfeld *et al*. 2018). The experimental amenability of worms makes them not just superb research subjects but also useful pedagogical tools. While excellent classroom additions to illustrate many biological processes, the educational potential of worms has lagged due to the expensive equipment required for their study. In most educational settings, lack of equipment discourages individual exploration and falls short of the promise owed to young and driven students. One of the places where this is felt strongly is in the automated quantification of animal behavior. Many systems have been developed over the years (and are currently used across the world) for the rapid and unbiased quantification of behavioral phenotypes. For example, we used tracking systems to compare the ability of mutant *C. elegans* strains to transition between gaits (Vidal-Gadea *et al.* 2011), and Deng and colleagues used them to study the role of inhibitory GABAergic motorneurons during rapid locomotion (Deng *et al.* 2020, see Husson *et al.* 2012 for a review of the use of tracking systems in *C. elegans*). Until recently, the automated quantification of *C. elegans* behavior was only feasible in specialized labs. Recent advances have begun to reduce the expense and complexity of automatically quantifying animal behavior. For example, the Haspel lab at NJIT made use of the recently developed Tierpsy behavioral software (Avelino *et al.* 2018) to build numerous animal tracking systems that are user friendly, and able to achieve levels of kinematic analysis that previously required considerably more expensive setups (Deng *et al.* 2020).

After building and witnessing the ease of use and power of the setup described above, we decided to reverse-engineer a similar worm tracker system for classroom deployment. Our intention was to develop a bare-bones system that allowed as many students as possible to engage in individualized research projects. We implemented this worm tracker in an introductory Cellular and Molecular Laboratory course at Illinois State University (BSC220). In this class, small groups of (three) students built and regularly used the trackers to film and quantify the behavioral phenotypes of *C. elegans* worms illustrating different cellular or genetic manipulations. Because lab fees associated with molecular labs can be restrictive, a key consideration in our approach was cost reduction (along with ease of build and use). Here we describe an affordable (~US$30) worm tracking system whose construction and use is amenable to high school and undergraduate students. We provide a list of materials, step by step assembly instructions, and illustrate its ease of construction and use to measure kinematic differences between wild-type and mutant *C. elegans* worms.

The process of filming and analyzing animal behavior involves three distinct components. First is the construction of a worm tracking system, which is the focus of this manuscript. The worm tracking system needs to be able to image nematodes at sufficient magnification and contrast to allow machine vision algorithms to locate and track individual animals. While inexpensive USB microscopes are available for purchase (and indeed are used in this build), one key feature of useful worm trackers is their ability to provide optimal underneath illumination in order to produce homogeneous, high-contrast, images. The second component of the system consists of an image acquisition program used to film the animals. There are several free programs available including micro-manager, Widows Camera, and the programs distributed with the USB microscopes, such as the one used in our tracker. The third and final component of the ensemble is a worm tracking program able to detect and track individual nematodes from a movie or series of images. Presently Tierpsy is an outstanding free kinematic package with widespread use among worm labs (Javert *et al*. 2018). However, because of its restrictive system requirements, we opted for a more limited but easier to install (and use) ImageJ plugin named wrMTrck (Nussbaum-Krammer *et al.* 2015). The wrMTrck plugin extracts many useful parameters such as animal size, velocity, and body bend frequency.

## Methods

**Worm Tracker Construction**

Refer to Figure 1A for a series of images illustrating assembly steps described below. Numbers (1-14) were used to refer to steps in Figure 1, while letters (A-I) were used to refer to the stencil parts (Extended Data Figure).

1) Print and glue the stencil (Extended Data Figure) to a sheet of corrugated cardboard (thicker cardboard is harder to cut but provides a stronger build).

2) Use an x-acto knife (or scalpel) to cut the pieces to be used in the tracker following directions on stencil.

3) Pre-fold pieces (where indicated), begin assembly by folding the tracker base (labelled A on the stencil).

4) Glue the USB light braces (B) to secure the USB light beneath the tracker, making sure the light is aligned with the circular hole on the base, and that the LED faces upwards (through the hole). The USB plug from the LED light should protrude through the rectangular hole cut in the back of the base.

5) Use glue on (C) to close the base up.

6) Pre-fold and glue the scope towers (D) along the longest sides of the base.

7) Glue the tower brace strip (E) to the top of both tower arms to provide structural reinforcement.

8) Cut and glue the worm plate holders (F) onto the tracker base.

9) Cut a piece of diffuser cloth and glue it to the diffuser holder (G) (a Kimwipe or tissue paper will serve as a diffuser if fabric is not available).

10) Wrap the inner microscope holder (H) tightly around the top of the USB scope and glue it onto itself, creating a tight sleeve for the scope. Make sure to leave the USB light dial (above) and the focus dial (below) on the microscope free to rotate.

11) Fold each of the outer scope holders (I) in half, gluing the ends to the inner holder.

12) Insert the folded ends of the microscope holders through the cut in the towers; they should be tight but allow vertical adjustment.

13) Connect the USB light to a USB LED dimmer, and this to a USB power outlet. The USB dimmer allows control of the amount of light reaching the setup which is important to obtain homogeneous illumination.

14) Place a 5 cm petri dish with nematodes on the plate holder and connect the microscope to a windows PC with preinstalled acquisition software, ImageJ, and the wrMTrck plugin to start using the setup.

Depending on the materials used and number of units built, the instructions above will produce worm tracking systems costing as little as US$30 (see list of parts below). We feel that this is a permissive cost in the context of most secondary and tertiary educational settings. Several upgrades can be implemented that increase the durability (and cost) of the tracker. For example, replacing the cardboard with plastic or wood would produce a much stronger and longer lasting device. For the sake of minimizing difficulty and cost of the build we show here the most inexpensive and simplest configuration. The tracker described here was successfully constructed and used by our BSC220 students and by high school interns in our lab.

**Image Acquisition**

Once the worm tracking system is assembled and connected to a windows machine, the acquisition software provided by the microscope manufacturer can be used to film freely behaving animals. For the example presented here, we used *Windows Camera*, which comes preinstalled in all Windows PCs. We should mention that the tracking software (ImageJ) is capable of importing a wide array of image and movie formats. However, depending on the software used, the user might need to convert their image or movie files into one of the standard formats used by ImageJ (e.g. TIFF and Jpeg images, AVI movies, etc.). Once the software is started, the user simply needs to select the camera and select the filming option on the control ribbon. The US$20 USB microscope we tested comes with a calibrating ruler (used to establish the magnification), and with an Android phone connector, which enables students owning this type of device to record movies without the need for a computer. Before filming, place the diffuser slide below the plate of worms. The diffuser ensures that the LED light is evenly distributed throughout the plate. Uniform lighting is of paramount importance during filming. An agar plate with worms to be filmed is then placed above the diffuser. It is best to not attempt filming too many worms at one time, as this increases the likelihood of animals colliding (which complicates tracking). In our experience 6 to 8 worms is ideal. A copper ring can be placed on the plate to keep all the animals within the field of view (Simoneta and Golombek 2006). We usually film through the lid of the plate, but this can be removed if reflections appear on its surface. We never use the LED lighting that comes onboard with the USB microscopes. This type of illumination (from above) is not optimal when filming transparent worms, as it results in very poor contrast. The microscope has a red button that allows the user to switch between focus and magnification when turning the silver dial present. The focus dial on the microscope is used to bring animals into focus. The LED light installed underneath the tracker can be controlled using the LED dimmer connected to it. Optimal results are obtained when plates are devoid of any visible imperfections that might challenge the animal detection plugin. To compare worm strains with different locomotor abilities we used eight animals and filmed for 60 second movies at 25 fps. Healthy wild-type worms move at about 1 cm/min using body bends ranging in frequency between 0.5-2.5 Hz depending on the substrate (Vidal-Gadea *et al.* 2011). Therefore, the parameters used by our setup are appropriate for most rudimentary analyses. When filming, we recommend making multiple movies in anticipation of possible challenges during the analysis step.

**Animal Tracking**

Once the animals have been filmed and avi files generated, they can be imported into the analysis software (ImageJ) to track the worms and generate descriptive statistics. As mentioned above there are many available tracking packages, including a few freely available ones. For use in a research lab setting, Tierpsy is likely the best option providing a plethora of useful parameters that include centroid-based metrics (e.g. velocity), and posture-based metrics (e.g. body curvature). An alternative, that is more amenable to a high school or undergraduate lab setting, is the wrMTrck plugin for ImageJ written by Jesper Pedersen (Nussbaum-Krammer *et al.* 2015). Among the useful parameters measured by this plugin are body lengths travelled per second, body bend frequency, speed, and animal size. These metrics are sufficient to distinguish populations that perform differently due to a plethora of possible experimental manipulations.

**Evaluation of the tracking system**

***Camera.*** To evaluate our system’s capabilities, we filmed worms under different conditions. The camera manufacturer manual indicates the scope is capable of magnifications between 40X and 1,000X, up to 30 fps, and 5 megapixels resolution. Magnification can be controlled using a dial on the scope (this also requires changing the height of the scope). The maximal system resolution we achieved was 640×480 pixels, and the maximal frame rate was 25 fps. At maximal magnification, our field of view was 1.2 mm wide (or 533.3 pixels/mm). The focus dial present mid-unit becomes a zoom dial when the red zoom button on the unit is depressed. Our tracker has vertical slits which allow changes in magnification. Operating at maximal magnification and speed, the videos obtained by the system are suitable to study various commonly measured physiological processes in worms including digestion, defecation, egg laying, and pharyngeal pumping in single adult animals. At the lower magnification range, we could film populations of worms with a visual field 10mm wide (80 pixels/mm). This resulted in adult worms measuring an average of 86 pixels in length. Tierpsy is the software package commonly used in professional settings and requires worms to measure a minimum of 100 pixels in length to obtain a full kinematic readout of animal postures. At intermediate magnifications our system achieves this benchmark, opening the door for using these movies for more in-depth analysis than what is available with the (easier to use) wrMTrck plugin for ImageJ.

***Tracking.*** We filmed different *C. elegans* strains crawling and swimming. We next processed the avi movie files using the wrMTrck ImageJ plugin as described by the authors (Nussbaum-Krammer *et al.* 2015). To assess how accurate the plugin was at tracking worms in our movies, we multiplied the number of frames in each movie by the number of animals present in each movie. This allowed us to generate the maximum possible number of tracked events in each experiment. For example, a 60 second movie of 8 worms filmed at 25 fps would theoretically result in a maximum of 12,000 counted events (60 sec * 8 animals * 25 fps = 12,000). We divided the total number of events detected by wrMTrck by the theoretical maximal to generate a quotient of detection (QD) which ranges between 1 (when all animals are tracked the entire movie) to 0 (when no animal is tracked throughout the whole movie). Analyzing movies made with our tracker wrMTrck had a QD= 0.71+/-0.14SD). In our experience, this quotient is similar to those obtained by professional software packages. The majority of the tracking error is the result of animals temporarily contacting the edge of the frame, momentarily exiting the field of view, or animals colliding forcing their outlines to merge. These conditions cause the plugin to ignore animals (a common challenge for many machine vision algorithms). To illustrate the performance of the system, we included a video (Extended Data Video) which illustrates the successive steps of image processing and tracking for a group of swimming *unc-31(e928)* mutants. The tracking for this example had a QD of 0.84 resulting from occasional animal outlines merging. wrMTrck calculates many useful parameters such as frequency (body bends per second), and normalized velocity (body lengths per second) that do not require an animal to be continuously tracked for the duration of the video. In the example provided below we report swimming frequency and normalized velocity for the weighted average of the tracks obtained from each movie. One limitation of the wrMTrck plugin arises from the way the algorithm uses posture ratios to calculate body bend frequency. The lateral to longitudinal ratio used to detect body bends makes this parameter accurate only when analyzing swimming behavior when the posture of the worm forms a single body wave at the time. During crawling multiple waves travel posteriorly down the worm’s body and the algorithm consistently overestimates the number of body waves produced.

***Cost-benefit.*** The durability of the worm tracking system can be increased by investing in stronger materials. We focused on producing a device that could be easily assembled, is inexpensive, and could last at least one semester of regular classroom use. Because of this, our system is not amenable to repeated changes of magnification settings. These changes are achievable using the vertical microscope slits that permit sliding the microscope up or down. We recommend having a couple of units dedicated to either high or low magnification if this is something the user anticipates needing regularly. Even with repeated changes in magnification, we note that our builds made with thick corrugated cardboard lasted an entire semester in our undergraduate lab course. We also note additional limitations of our system compared to more expensive counterparts. More expensive (>US$1,500) systems like the one in use at the Haspel lab, use infrared light and cameras to avoid potentially disrupting behavior. The cameras in such systems are high resolution and are able to obtain high frame rates (>50 fps), wider fields of view (>3 cm), and high spatial resolution (>1,900 pixels). These advantages allow users to film a hundred or more animals in a 5 cm petri dish while still obtaining the high resolution needed by the Tierpsy worm spining algorithm. The larger field of view of these more expensive systems allows the performance of behavioral assays (e.g. chemotaxis assays). Although our system cannot capture complex behaviors like chemotaxis, it is able to perform basic locomotor measurements in a classroom setting.

**Sample Application**

To illustrate the use of the worm tracking system, we compared day one adult wild-type (N2) *C. elegans* worms to day 1 adult *unc-31(e928)* mutants. UNC-31 is expressed in synapses and predicted to have calcium binding activity. Loss of function mutations in this gene result in impaired chemical synaptic transmission, and severe locomotor defects (Speece *et al.* 2007). We set up our assays by placing eight animals inside a copper ring (1 cm across) on an unseeded NGM plate. Copper repels worms and prevents animals from exiting the field of view during the assay (Simoneta and Golombek 2006). We next flooded the copper ring with 50 µl of liquid NGM to induce swimming. After two minutes of acclimation, we used the tracker to film worms for 60 seconds at 25 fps using our worm tracking system (Figure 1B). We repeated the experiment four times for each condition using four different sets of animals. Movies were exported as uncompressed AVI files (UYVY codec) and uploaded to ImageJ. We used the “set scale” function in ImageJ to enter the appropriate magnification. We next used the scroll bar on the movie window to establish the limits of maximal vertical and lateral worm excursions. We used the rectangular area of interest tool to select a region large enough to include the trajectory of all the animals during the entire movie. We cropped the video to this smaller area to reduce file size (and exclude the copper ring or other visual artifacts). We then converted the movie to 16bit. Following wrMTrck instructions, we generated a maximum intensity z projection picture of the movie. Next, we used the image calculator function of ImageJ to find the difference between the maximum projection and the movie. This removed the background from the movie and left only the moving objects (worms). In the final pre-processing step, we adjusted the threshold of the movie image using the suggested maximum entropy setting and moving the sliders to ensure only worms remained selected. We scrolled through the movie to ensure that the thresholding setting remained accurate throughout the movie. When applying the threshold, we selected the option “dark” background in order to produce a new movie with dark worms in a light background, which is what wrMTrck requires. We next performed some initial measurements on the original movie file, in order to establish the size of worms in pixels (as this is what the plugin uses in its measurements). After this, we were ready to apply the wrMTrck plugin. Running the wrMTrck plugin we obtained a table with measurements and several other useful quality control features, such as a movie with the tracked objects, and a bend track plot which allows the empirical determination of the bend threshold to be used in bend cycle determination. Please see Extended Data Video for a demonstration of the previous steps.

We followed the processing described above for the N2 and *unc-31(e928)* strains and then focused on the body bends per second (i.e. swimming frequency), and body lengths per second (swimming velocity normalized to size), as these parameters are accurate descriptors of locomotor performance. The algorithm terminates a track whenever animals intersect, or whenever they contact the edge of the visual field. Therefore, there are more tracks than animals. Averaging the tracks would result in animals with fragmented trajectories being overrepresented. We therefore calculated a weighted average for each parameter of interest. To do this, we took each track, multiplied the number of frames it lasted by the average measure reported for the parameter of interest (e.g. body bends per second). We then summed the products for all the tracks in the movie and divided this by the total number of frames tracked (for all animals) in the movie. The resulting value is the weighted average for the parameter in question. It assigns each contributing track a weight proportional to the fraction of the movie it lasted (i.e. shorter tracks having less weight than longer ones). We then plotted these values and used two-tailed t-tests to compare the weighted averages of four assays of each strain for normalized swimming velocity and frequency. As previously shown (Speece *et al.* 2007), *unc-31(e928)* animals have severe locomotor impairments compared to wild types (Figure 1D and E respectively).

**Concluding remarks**

We described a worm tracking system that introduces an inexpensive, easy to construct and operate device. While intended for classroom use, it can deliver results approaching those of more expensive professional systems. It is versatile enough to automatically measure common locomotor parameters, and to allow the study of physiological processes such as pharyngeal pumping, defecation, and egg laying which require high magnification and contrast. Although we did not test the system with other species, the system is likely useful for tracking other small organisms such as arthropod larvae, or even paramecia. We believe that the affordability and ease of use of the system will make it a useful addition to classrooms where physiological and behavioral consequences of gene and cellular activity are taught or investigated.

## Reagents

***Organisms***

We used 32 adult (day 1) wild-type (N2) *C. elegans* worms, and 32 *unc-31(e928)* adult (day 1) mutant worms (CB928) obtained from the Caenorhabditis Genetics Center (CGC), which is funded by the NIH Office of Research Infrastructure (P40 OD010440). Animals were raised under standard lab conditions (Brenner 1974) at 20°C and fed on OP50 *E.coli* until used.

***Statistics***

We used SigmaPlot 11(Systat) two-tailed t-tests to compare the swimming velocity and frequency for both N2 and *unc-31(e928)* worms using the weighted averages for four replicates with eight different animals per assay.

***Hardware***

The following hardware was used to construct the worm tracking system:

A Windows computer may be required for filming, and it is required for analysis (not included in cost calculation).

USB microscope. US$20. Can be obtained from Amazon (ASIN: B08BFJZJ6N)

USB LED light. US$9. One needed (but comes in 8-pack) from Amazon (ASIN: B088KPZYS9)

USB LED dimmer. US$9. Suggested. Can be obtained from Amazon (ASIN: B01I17OHVQ)

Diffuser fabric. US$8. Enough for many setups. Can be obtained from Amazon (ASIN: B01JGY8U66)

Total build US$46 (US$31/ea if eight units are built)

***Software***

For use with the USB microscope there are many freely available software including Plugable Digital Viewer: https://plugable.com/drivers/microscope/. For this manuscript we used Windows onboard Camera software. There are differences in the level of control afforded by each software package. We recommend the user experiment with a couple free programs to determine which one suits their needs best.

To run the worm tracking software, it is necessary to first install ImageJ which is an open source Java image processing program found here: https://ImageJ.net/Fiji/Downloads We have used a few different versions of the program (ImageJ 1.53e and ImageJ 1.52p) without problems.

Worm tracking in ImageJ is accomplished by the wrMTrck plugin created by Jesper Pedersen available here: http://www.phage.dk/plugins/wrmtrck.html. A useful set of written and video tutorials are available online here: (http://www.phage.dk/plugins/download/wrMTrck.pdf and here: https://www.youtube.com/watch?v=V9M0s0E-0uI respectively.
